# The C-Fern (*Ceratopteris richardii*) genome: insights into plant genome evolution with the first partial homosporous fern genome assembly

**DOI:** 10.1038/s41598-019-53968-8

**Published:** 2019-12-03

**Authors:** D. Blaine Marchant, Emily B. Sessa, Paul G. Wolf, Kweon Heo, W. Brad Barbazuk, Pamela S. Soltis, Douglas E. Soltis

**Affiliations:** 1 Present Address: Department of Biology, Stanford University, Stanford, CA 94305 USA; 2 Department of Biology, University of Florida, Gainesville, FL 32611 USA; 3 Florida Museum of Natural History, University of Florida, Gainesville, FL 32611 USA; 4 The Genetics Institute, University of Florida, Gainesville, FL 32611 USA; 5 Department of Biology, Utah State University, Logan, UT 84322 USA; 6 Present Address: Department of Biological Sciences, University of Alabama in Huntsville, Huntsville, AL 35899 USA; 7 Department of Applied Plant Sciences, Kangwon National University, Chuncheon, 24341 Korea; 8 The Biodiversity Institute, University of Florida, Gainesville, FL 32611 USA

**Keywords:** Molecular evolution, Plant evolution, Genome duplication

## Abstract

Ferns are notorious for possessing large genomes and numerous chromosomes. Despite decades of speculation, the processes underlying the expansive genomes of ferns are unclear, largely due to the absence of a sequenced homosporous fern genome. The lack of this crucial resource has not only hindered investigations of evolutionary processes responsible for the unusual genome characteristics of homosporous ferns, but also impeded synthesis of genome evolution across land plants. Here, we used the model fern species *Ceratopteris richardii* to address the processes (e.g., polyploidy, spread of repeat elements) by which the large genomes and high chromosome numbers typical of homosporous ferns may have evolved and have been maintained. We directly compared repeat compositions in species spanning the green plant tree of life and a diversity of genome sizes, as well as both short- and long-read-based assemblies of *Ceratopteris*. We found evidence consistent with a single ancient polyploidy event in the evolutionary history of *Ceratopteris* based on both genomic and cytogenetic data, and on repeat proportions similar to those found in large flowering plant genomes. This study provides a major stepping-stone in the understanding of land plant evolutionary genomics by providing the first homosporous fern reference genome, as well as insights into the processes underlying the formation of these massive genomes.

## Introduction

There are estimated to be over 400,000 species of extant land plants^1^, encompassing an enormous array of morphological, physiological, and ecological diversity. Accompanying this diversity is extraordinary variation in genome size^2,3^, spanning a 2,500-fold range from the bladderwort *Genlisea aurea* (~60 Mbp)^4^ to that of the monocot *Paris japonica* (150 Gbp)^5^. How these genomes are chromosomally partitioned also varies immensely, as land plants span a 360-fold range in chromosome number, from *2n* = 4 in *Haplopappus gracilis, Brachychome dichromosomatica, Zingeria biebersteiniana*, and *Colpodium versicola* to *2n* = 1,440 in the fern *Ophioglossum reticulatum*, the highest number reported for any eukaryote^3,6,7^. Understanding the processes underlying this enormous breadth in both genome size and chromosome number has long been a major area of interest among evolutionary biologists. However, sampling biases towards smaller, less complex genomes (e.g., *Arabidopsis*: 135 Mbp, *n* = 5) and crops have pervaded plant genome projects. Fortunately, recent technological advances have enabled the assembly and analysis of large genomes, such as those of conifers^8–10^, providing novel insights into the processes underlying genome and chromosomal composition. Despite this progress, the large genomes typical of homosporous ferns remain uninvestigated.

Polyploidy, or whole-genome duplication (WGD), is the traditional explanation for the large genomes and numerous chromosomes found in many plants, as WGD results in the complete doubling of the genome^11,12^. Among flowering plants, phylogenetic and genomic studies have identified WGD events preceding key radiations, such as those of the core eudicots (~70% of the flowering plants)^13^, monocots^14^, and the entirety of flowering plants^15–17^. In addition, it was demonstrated that even species with minute genomes, such as the carnivorous plant *Utricularia gibba*, with *n* = 13 and a genome size of 80 Mbp, have experienced multiple WGD events; *U. gibba* has undergone at least three ancient WGD events in the last 80 million years^18^. Thus, genome size and chromosome number may not be reliable indicators of WGD. This disparity between genome size and chromosomal composition relative to WGD frequency has altered our understanding of genome evolution as the question has changed from whether or not an organism is polyploid, to how many rounds of polyploidy an organism or lineage has experienced in its evolutionary history.

Although a few fern genomes, such as those of the heterosporous water ferns (<1% of fern diversity), are less than 250 Mbp^19^, the average homosporous fern genome is 12 Gbp, nearly five times the size of the genome of maize (2.5 Gbp) and over 80 times that of *Arabidopsis*. In addition, homosporous ferns typically have substantially more chromosomes than seed plants, with an average haploid chromosome number of 59 compared to 16 in flowering plants or 12 in gymnosperms^3,20^. As a result, longstanding hypotheses have proposed that multiple, repeated WGD events were the major factor contributing to the high chromosome numbers and large genomes of ferns^20–22^.

It was originally hypothesized that homosporous ferns undergo intense selection favoring polyploidy to buffer against a putatively high rate of inbreeding that results from their unique life history^20,23^. The homosporous fern life cycle includes a free-living haploid gametophyte phase with the potential for intragametophytic selfing (IGS)^23^, or gametophytic selfing sensu Haufler *et al*.^24^ – a process that can produce a completely homozygous diploid plant in a single generation and thus expose any deleterious mutations. However, numerous isozyme analyses demonstrated that fern species with the lowest chromosome numbers within a given genus (ranging from *n* = 27 to 52) were functionally diploid, producing typical diploid numbers of isozyme loci rather than multiple loci as seen in truly polyploid species with multiples of these low chromosome numbers^25–27^. Despite the lack of isozyme evidence for repeated polyploidy in diploid fern species, multiple copies of chlorophyll a/b-binding protein genes were discovered in the diploid fern *Polystichum munitum*, but the duplicated genes were nonfunctional^28^. Furthermore, early population genetic investigations showed that homosporous ferns have highly variable mating systems and are typically outcrossing, refuting the hypothesized force (intense inbreeding depression via IGS) driving selection for polyploidy^29^. More recently, a genetic linkage map showed that *Ceratopteris richardii* has one of the highest proportions of duplicated loci among plants (76%) yet lacks large, duplicated blocks that would be indicative of polyploidy^30^. In addition, a paralog-age distribution analysis of *Ceratopteris* estimated an ancient polyploidy event over 180 million years ago (mya); however, the data used for this analysis were from a shallow EST library^31^. Despite little evidence for ancient polyploidy in ferns, chromosome count models suggest that 31% of fern and lycophyte speciation events involve WGD, compared to 15% in flowering plants^32^. However, these estimates of WGD refer to relatively recent polyploidy events (neopolyploidy) evident from chromosome numbers rather than ancient (paleopolyploidy) events deep in evolutionary history.

Ferns are also the only major lineage of land plants with a significant positive relationship between genome size and chromosome number, suggesting that fern chromosomes are relatively static compared to those of angiosperms and gymnosperms for which no such correlation exists^33,34^. While repeated episodes of WGD followed by extensive silencing and rearrangement cannot be discounted as an explanation for the paradoxical genomic, genetic, and chromosomal composition of ferns^21^, alternative processes underlying their large genomes and high chromosome numbers must be explored. Most notable among these alternative explanations for the large genomes of ferns is the impact of transposable elements (TEs) on genome size, as TEs make up the majority of genome space in a variety of eukaryotic lineages. For example, TEs are responsible for the difference in genome size between cultivated rice (*Oryza sativa*, 390 Mbp) and wild rice (*O. australiensis*, 965 Mbp)^35^. Phylogenetic reconstructions of major TE families in various plant lineages suggest that bursts of TE insertion result in inflated genome size^36–39^. However, genome inflation does not seem to be a one-way street, because unequal homologous recombination can eliminate repetitive regions, such as those produced by TEs^40,41^. Analysis of three conifer “giga-genomes” (20–30 Gbp) showed that these large genomes were derived not through WGD, but rather via extensive expansion of ancient TEs (especially retrotransposons) and an apparent inability to shed these repetitive regions via unequal recombination^8,42^. While TEs provide a possible alternative explanation for the large genome sizes of ferns as demonstrated in conifers, they cannot explain the high chromosome numbers of ferns. It is possible that ferns have ancestrally high chromosome numbers and a relatively low rate of WGD, yet this begs the question of how the high chromosome numbers were initially obtained. Aneuploidy or chromosomal fission are also possible explanations for the high chromosomal complement of most ferns^22,26,43^.

There are now hundreds of published flowering plant, gymnosperm, lycophyte, and bryophyte genomes, alongside the recent publication of two heterosporous water fern genomes^19^. While these water fern genomes, for *Azolla* and *Salvinia*, are much-needed references within the fern clade, they are atypical of 99% of ferns, in that these species are heterosporous and have very small genomes with few chromosomes (1 C = 0.25–1.76 Gbp, *n* = 9–22)^19^. To date, no sequenced genome is yet available for any homosporous fern^44^. This major information gap is made more startling when the high species diversity (>10,000 species), significant ecological roles, and economic importance of homosporous ferns are considered^45–50^. Due to their crucial phylogenetic position as sister to seed plants, ferns are key for investigating an array of both genomic and non-genomic traits and will permit a synthesis of genome evolution across seed plants^51,52^.

Here we investigated the genome of the homosporous fern *Ceratopteris richardii* (C-fern; 11.25 Gbp, *n* = 39), characterizing and classifying TE composition and assessing the extent of WGD. Our genomic data for C-fern, together with the recently published heterosporous water fern genomes^19^, help provide a genome evolutionary context not just for ferns, but also for all vascular plants. Collectively, these data will permit deductions about ancestral genome characteristics of seed plants and ferns, as in studies of other phylogenetically pivotal lineages^16,53^. Specifically, this *Ceratopteris* genome provides critical insights into the evolutionary genomics and paradoxes of the genomically long-neglected fern clade, in addition to serving as a valuable reference for future investigations into land plant genome composition and dynamics.

## Results

### Genome sequencing and assembly

Here we present the first sequence of a homosporous fern genome, providing a new resource for plant and evolutionary biology. The ability of homosporous ferns to undergo IGS (see above) partially simplified the assembly of this complex genome, as it made the sporophyte completely homozygous so that heterozygosity was not an issue in assembly. However, the quality of the *Ceratopteris* genome assembly and the computational resources required to assemble and analyze the genome reflect the technological difficulties of working with such a large and complicated genome with no closely related reference genome.

With paired-end short-read libraries totaling ~24X coverage from 1.8 billion cleaned reads, we assembled the 11.25 Gbp *Ceratopteris* genome into ~15 million contigs (>100 bp) or 988,403 scaffolds (>1,000 bp) (Table 1). We then combined and reduced the number of scaffolds using 8–10 Kbp mate-pair reads (13X coverage), producing a genome assembly (*CFern v1.1*) of 626,576 scaffolds with an N50 of 16 Kbp and total length of 4.25 Gbp, representing about 38% of the *Ceratopteris* genome.

**Table 1:** *Ceratopteris* genome assembly statistics.

Cytometric Genome Size	11.25 Gbp
Chromosome number	39
**Assembly V1.0**	
Meraculous Contigs	15,871,274 contigs
Total Size	4.21 Gbp
N50%	300 bp
Gaps	0
% GC	36
*CFern v1.0* (≥1,000 bp)	988,403 scaffolds
Total Size	2.69 Gbp
N50	3,376 bp
% Gaps	0.5
% GC	36
*CFern v1.1* (≥1,000 bp)	626,576 scaffolds
Total Size	4.25 Gbp
N50	16,289 bp
% Gaps	37
% GC	38
*CFern v1.1A* (≥10,000 bp)	133,755 scaffolds
Total Size	2.79 Gbp
N50	22,401 bp
% Gaps	44
% GC	38
*BAC.SubSample*	35 scaffolds
Total Size	3.03 Mbp
N50	97,182 bp
% Gaps	0
% GC	39

We also sampled a smaller portion of the *Ceratopteris* genome using long-read sequencing of 32 bacterial artificial chromosomes (BACs) of *Ceratopteris*. This subsample assembly (*BAC.SubSample*) only totaled 3 Mbp of the *Ceratopteris* genome (0.03%), but had an N50 of 97 Kbp, providing a small, but more accurate and contiguous sampling of the 11.25 Gbp genome as long-read technology is less biased by repeat elements and mis-assemblies. The GC content of *Ceratopteris* was 37.7%, very similar to that of both the gymnosperm *Picea abies* (Norway spruce) (37.6%) and the flowering plant *Amborella trichocarpa* (37.5%), yet lower than that of maize (46.9%), the liverwort *Marchantia polymorpha* (42.0%), and the lycophyte *Selaginella moellendorffii* (45.3%).

### Transcriptome assembly

From 12 PacBio SMRT cells, we obtained ~850,000 reads from which we produced 97,084 full-length, high-quality, cleaned transcripts (*IsoSeq.HQ*) ranging from 285 to 11,353 bp in length. When mapped onto the *CFern v1.1* assembly at 98% identity and 98% coverage, the *IsoSeq.HQ* transcripts were collapsed into 4,620 genes and 10,043 isoforms; however, when coverage was reduced to 50%, there were 11,924 genes and 23,278 isoforms. The 2.5-fold increase in identified genes and isoforms via reduced coverage shows that our scaffolds do not span entire genes in the majority of cases. To overcome this fragmentation and provide a set of high-confidence gene models, we implemented the Cogent genome-free protocol^54^ to produce 18,179 gene models (*UniCFernModels*) from the *IsoSeq.HQ* transcripts. Searching for 1,440 embryophyte single-copy orthologs^55^, we found 53% complete, 4.5% fragmented, and 42.6% missing.

### Polyploidy

**Figure 1:**
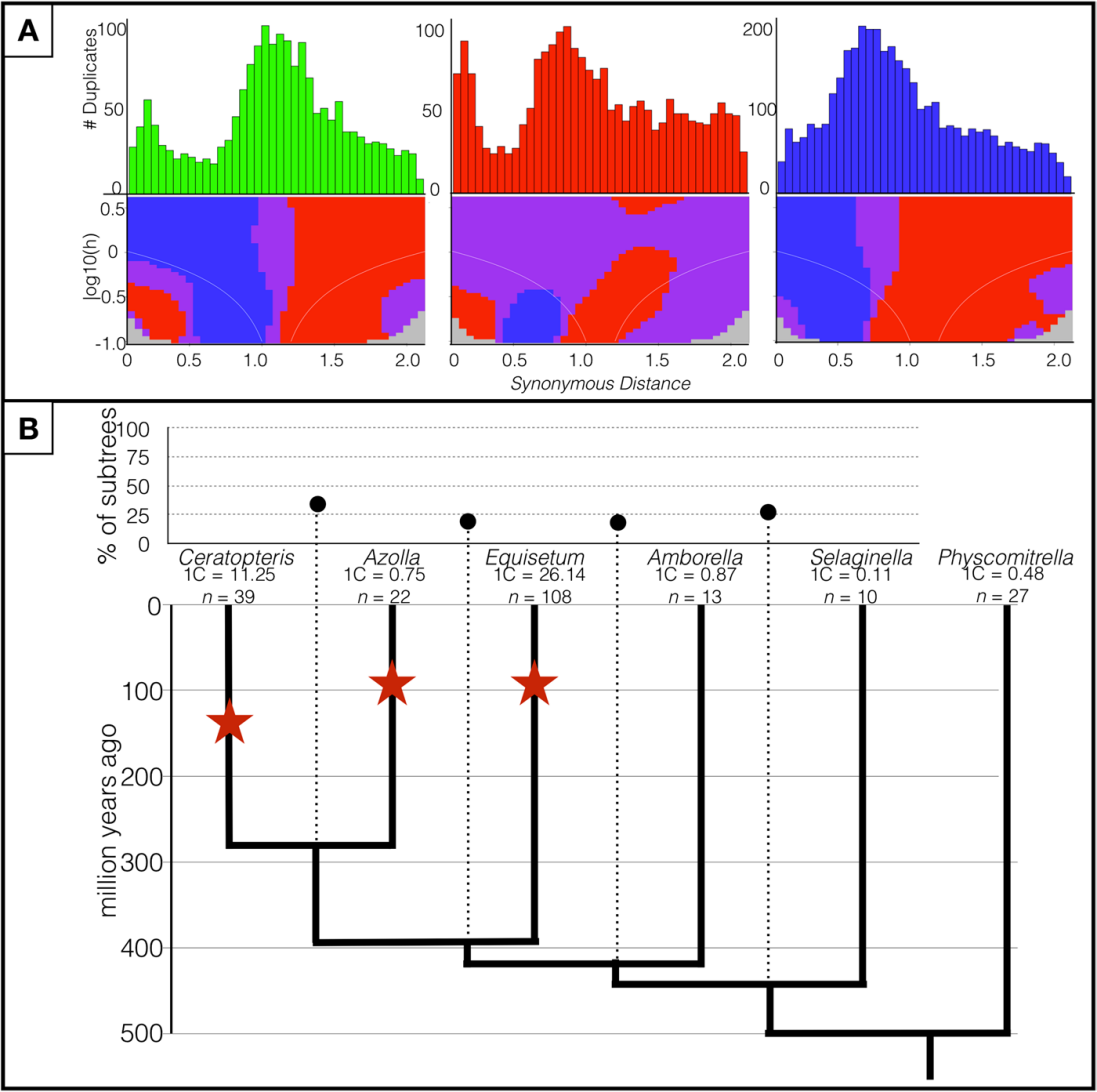
Polyploidy analyses of three fern species. (**A**) Paralog-age distribution analyses and associated SiZER plots of three fern species. Upper panels are *Ks*-based histograms (0.05 bins) of paralogs in *Ceratopteris richardii, Azolla filiculoides*, and *Equisetum giganteum*. Lower panels are SiZER plots of the above paralog-age distribution data and associated smoothing functions where blue indicates significant (α = 0.05) increases, red significant decreases, purple insignificance, and gray too few data points to determine. The white lines show the effective window widths for each bandwidth. Both upper and lower panels are on the same *x*-axis. (**B**) MAPS analysis across land plants and the associated WGD events (shown as stars). The percentages of subtrees that contain gene duplications shared by the descendent species of a given node are above the phylogeny (connected by dotted lines). Dates are based on Testo and Sundue^70^ and Morris *et al*.^67^.

To address the decades-old question of how common ancient polyploidy is in ferns, we employed sequence-based and cytogenetic approaches, which assessed three different temporal scopes of evolutionary history. Using paralog-age distribution analyses, we identified 1,800 paralogous gene pairs in the *UniCFernModels* with a *K*_*S*_ value between 0.1 and 2.1. A minor peak around *K*_*S*_ = 0.3 was detected; however, such small, “recent” peaks are often a result of small-scale gene duplications, not WGD^56^. In contrast, a single major peak was revealed in the synonymous distance plot of *Ceratopteris*, similar to those observed in *Azolla* and *Equisetum* (Fig. 1). Based on the significant transition from positive to negative in the SiZER plot, the *Ceratopteris* peak was at *K*_*S*_ = 1.1, compared to 0.8 in *Azolla* and 0.75 in *Equisetum*, similar to the original results found by Vanneste *et al*.^57^.

To determine whether the peaks found in these three ferns (*Ceratopteris, Azolla, Equisetum*) represent a shared WGD rather than three distinct WGD events, we used the Multi-taxon Paleopolyploidy Search (MAPS)^58^. We first recovered 10,182 orthogroups from the clustered amino acid sequences of *Ceratopteris, Azolla, Equisetum, Amborella, Selaginella*, and *Physcomitrella*. We isolated 4,836 orthogroups with amino acid sequences from all six species and estimated gene family trees for each orthogroup. Of the subtrees that fit the known fern topology, ((*Ceratopteris, Azolla), Equisetum*), 34% supported a gene duplication in the most recent common ancestor (MRCA) of *Ceratopteris* and *Azolla*, and 19% of subtrees fitting the ((*Ceratopteris/Azolla*, *Equisetum*), *Amborella*) topology supported a gene duplication shared across the three fern species (Fig. 1) – relatively low proportions compared to similar studies that identified shared WGD events^58^. These low proportions suggest three lineage-specific WGD events rather than one or two shared events between the three fern taxa.

While the previously described methods of data analysis for assessing WGD are appropriate at deeper time scales, both are susceptible to missing more recent WGD events. As mentioned above, relatively recent (close to zero along the *x*-axis) WGD in the *K*_*S*_ plots may be mistakenly attributed to small-scale duplications, while MAPS can only identify WGD events that have occurred prior to the MRCA of the next closest taxon included in the analysis. In the case of *Ceratopteris*, that would be 280 million years to the divergence of *Ceratopteris* and *Azolla*, thus only events older than that can be identified by MAPS.

**Figure 2:**
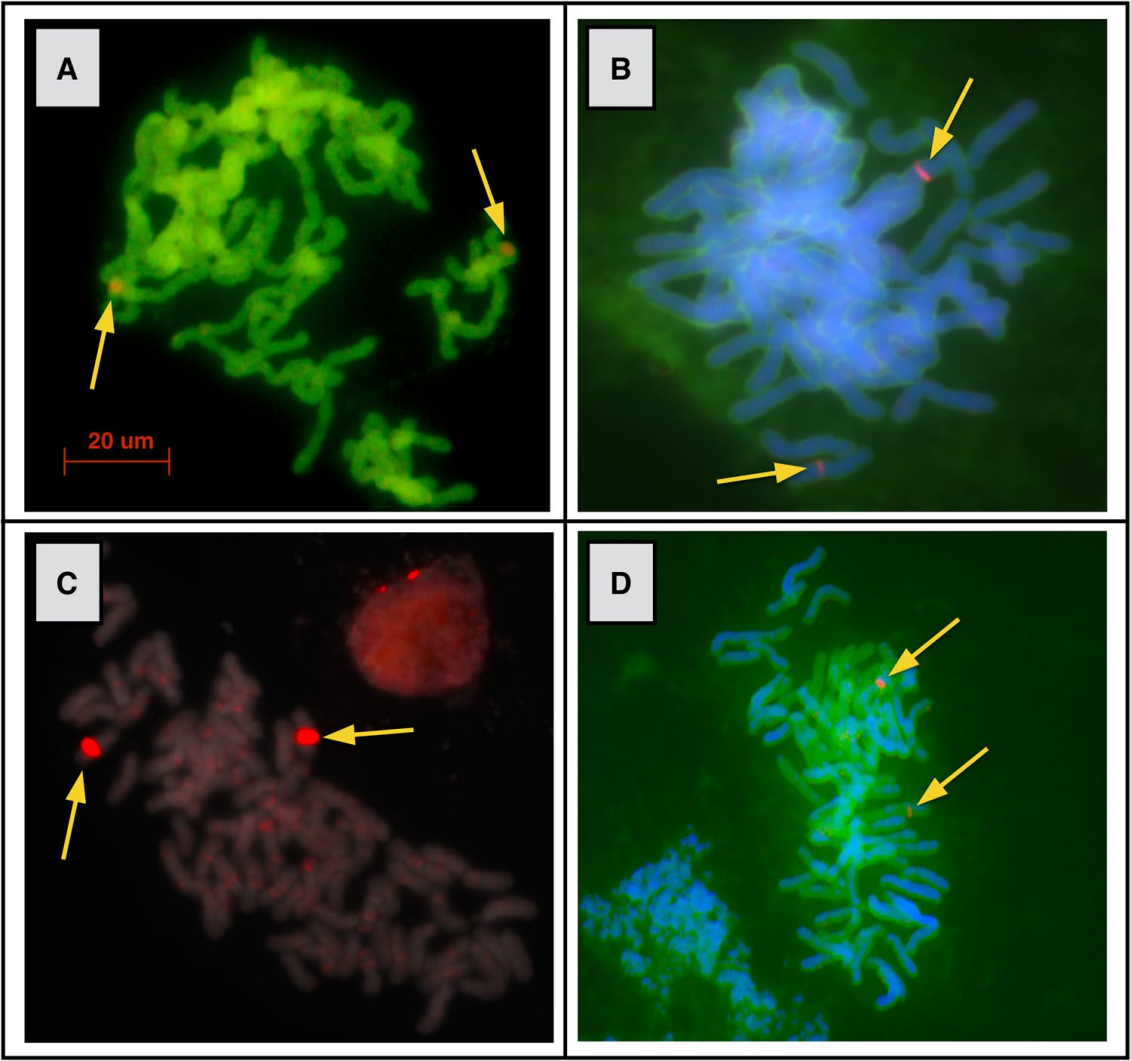
Fluorescent *in situ* hybridizations of *Ceratopteris* chromosome spreads. The fluorescent probes are of 100–150 Kbp DNA fragments from BACs of *Ceratopteris*. Primary “diploid” localizations (red bands labeled with arrows) are shown in all four panels, while weak secondary localizations, most likely reflecting repetitive elements, are apparent in (**C**); note scattered faint red staining in addition to the two strong primary signals. BACs are from wells A12 (**A**), B3 (**B**), A8 (**C**), and B9 (**D**) in Plate CR_Ba #624, Green Plant BAC Library Project, Clemson University Genomics Institute.

Our cytogenetic approach using FISH suggests the ploidy of an organism by localizing 125–150 Kbp BAC DNA fragments to the chromosomes where the DNA fragment is found. If the organism is diploid, only two localizations will be apparent, while a polyploid should have more than two localizations. BAC-FISH evidence of polyploidy is relatively short-lived as studies of *Nicotiana* allopolyploids found that five million years after the WGD event, the two parental genomes in the polyploid were no longer distinguishable due to genome turnover, mutations, and small-scale duplications^59^. However, our BAC-FISH results further corroborated our sequencing results in demonstrating a lack of evidence for recent WGD in *Ceratopteris*. Significantly, we detected only two primary localizations of each BAC probe we exposed to the *Ceratopteris* chromosome preparations (Fig. 2), suggesting diploidy. In a few cases, weak secondary localizations, or “ghost bands,” were found on multiple chromosomes; however, these are likely a result of repeat elements that are distributed throughout the numerous chromosomes.

### Repeat diversity

**Figure 3:**
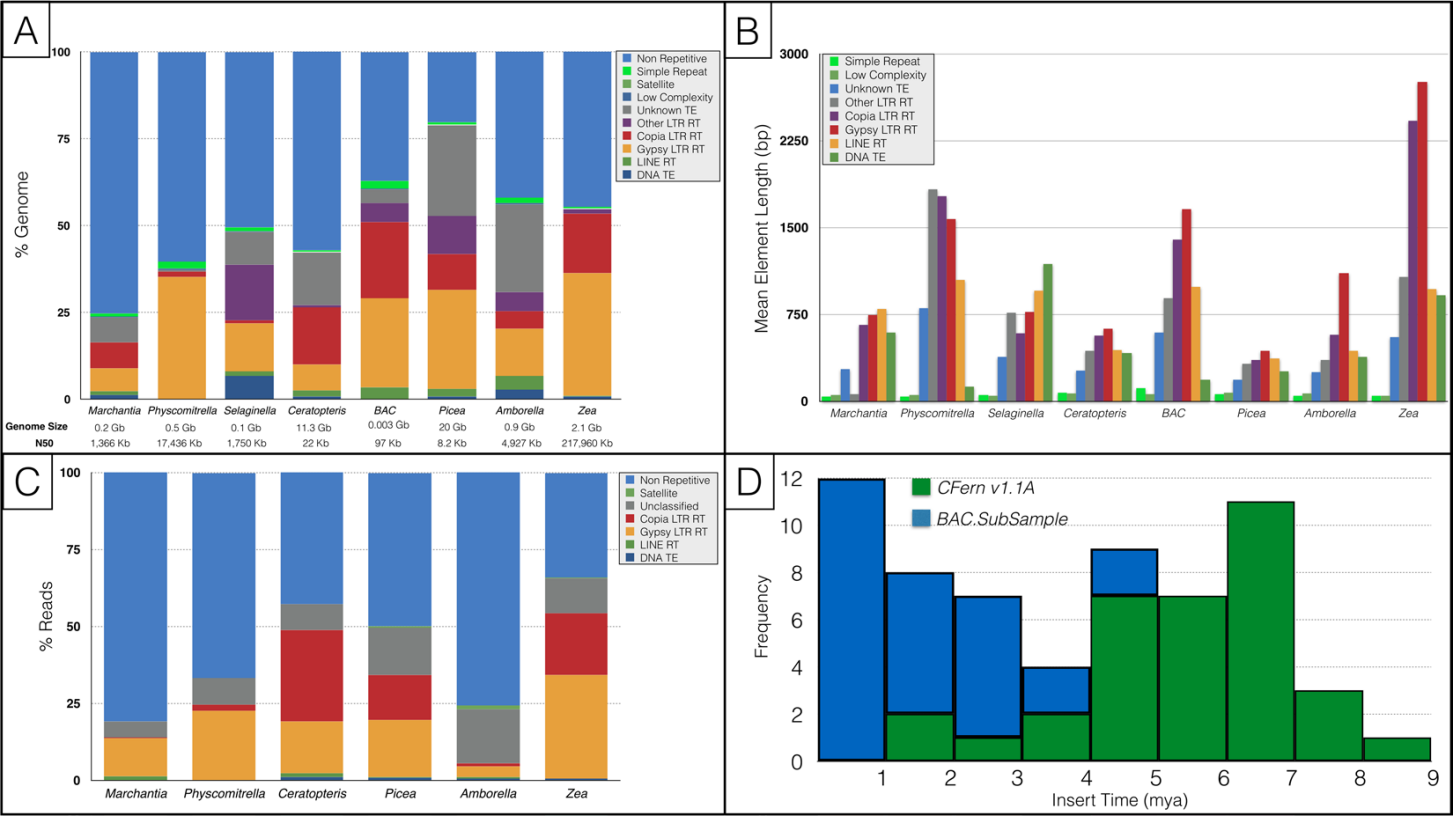
Repeat composition, lengths, and insertion timing for representative embryophyte genome assemblies. (**A**) Genome proportions of repetitive and non-repetitive elements for seven taxa spanning land plants, as well as *BAC.SubSample*, using genome-based analyses. Genome sizes and N50s for analyzed genome assemblies are also provided. (**B**) Mean repeat element lengths based on genome assembly analyses (**A**) for seven embryophyte taxa and *BAC.SubSample*. (**C**) Genome proportion of repetitive and non-repetitive elements using read-based clustering analyses^111^. (**D**) LTR RT insertion dates in *Ceratopteris* based on the *CFern v1.1A* and *BAC.SubSample* assemblies. Insertion dates were inferred from the similarity of long terminal repeat regions of the LTR RTs and a neutral substitution rate of 6.5 × 10^−9^ per site per year.

In total, ~42% of the *CFern v1.1* *A* assembly was composed of repeat elements (Fig. 3a). The *Copia* LTR RTs were the most prolific with over 800,000 elements making up 16.5% of the assembled genome, followed by the *Gypsy* LTR RT superfamily with 330,000 elements and accounting for 7.5% of the genome (Table 2). In comparison, Class II DNA transposons include members of 17 different super-families, yet only totaled 52,000 elements and <1% of the genome. The LINE RTs similarly covered 1.6% of the genome across 64,000 elements. Low-complexity, satellite, and simple repeats all covered <0.5% of the genome.

**Table 2:** *Ceratopteris* repeat diversity and composition.

Class	Order	Superfamily	Element count	Length (bp)	% Genome
Retrotransposon	LINE	Uncategorized	1458	523887	0.02
		RTE-BovB	421	128815	0.00
		Jockey	434	127827	0.00
		R1	2565	1241869	0.04
		RTE-X	8733	3937956	0.14
		L2	19047	3652063	0.13
		L1-Tx1	23635	15522256	0.56
		L1	47105	20464731	0.73
	LTR	Uncategorized	23507	7859618	0.28
		DIRS	361	45453	0.00
		Pao	1494	462814	0.02
		Gypsy-Troyka	5331	3393861	0.12
		ERV1	8083	6191165	0.22
		Gypsy	329706	207014935	7.42
		Copia	812470	460237954	16.50
DNA Transposon		Uncategorized	3289	693293	0.02
		hAT-Tip100	374	251768	0.01
		CMC-Mirage	416	153347	0.01
		MULE-MuDR	425	41133	0.00
		TcMar	530	83430	0.00
		hAT-hATw	622	343180	0.01
		Harbinger	627	224160	0.01
		PiggyBac	1230	122417	0.00
		Dada	1981	1130399	0.04
		CMC-EnSpm	2339	1276711	0.05
		Sola	2739	1565090	0.06
		hAT	2774	819873	0.03
		Maverick	4082	2443709	0.09
		hAT-Ac	4625	1727566	0.06
		PIF-Harbinger	4982	1214259	0.04
		En-Spm	10115	5487072	0.20
		hAT-Tag1	15720	6076071	0.22
		Helitron	4260	812693	0.03

The repeat content and percent coverage were considerably higher in the long-read *BAC.SubSample* assembly (63%). Nearly 26% of the subsample assembly was made up of *Gypsy* LTR RTs and 21.8% was *Copia* LTR RTs, while the LINE RTs and DNA transposons represented 3.2% and 0.16%, respectively. Low-complexity and simple repeats made up 0.2% and 2.2% of the *BAC.SubSample*, respectively. The mean lengths of all of the repeat types in the *BAC.SubSample*, with the exception of the DNA TEs, were more than double those of the *CFern v1.1* *A* assembly, and the *Copia* and *Gypsy* elements were nearly three times as large in the subsample compared to those of the *CFern v1.1* *A* assembly.

Similar to the *BAC.SubSample* results, read-based analyses of *Ceratopteris* also estimated that ~60% of the *Ceratopteris* genome is repetitive with 17% in *Gypsy* elements and 30% in *Copia* elements (Fig. 3c). The read-based analysis and *BAC.SubSample* characterization analysis are more accurate for assessing general genome composition compared to repeat characterization of the *CFern v1.1* *A* assembly, as they are not biased by the short-read assembly which can have trouble assembling repetitive regions beyond the length of the reads. This assembly bias is probably the reason the *CFern v1.1* assembly was limited in low-complexity, satellite, and simple repeat elements. This limitation is also apparent in comparing the mean lengths of the *CFern v1.1* *A* LTR RTs to those of the *BAC.SubSample* (Fig. 3b), as the latter assembly could likely span those longer repetitive regions via long-read technology.

We directly compared the repeat content of *Ceratopteris* to that of other land plants by applying the same assembly-based repeat characterization protocol to *Amborella trichopoda*^16^, a monocot (*Zea mays*)^60^, a liverwort (*Marchantia polymorpha*)^61^, a lycophyte (*Selaginella moellendorffii*)^62^, a conifer (*Picea abies*)^8^, and a moss (*Physcomitrella patens*)^63^. We chose to run our own analyses on these genome assemblies rather than comparing our results to those of previously published results due to the wide variation in repeat characterization analyses utilized. In addition, we ran the read-based clustering analysis on the above taxa, with the exception of *Selaginella*, using short reads from these assemblies covering 0.5X of each genome. Due to the previously mentioned limitations of the *CFern v1.1* *A* assembly, we largely focused on the *BAC.SubSample* and read-based results for comparing relative proportions of repeats to other taxa.

Compared to six other land plant genome assemblies, the *BAC.SubSample* was second in repeat proportion behind only *Picea* (Fig. 3a). The *BAC.SubSample* had proportions of *Copia* elements similar to those of *Zea*, substantially higher than any of the other genomes analyzed. The other super-family of LTR RTs, the *Gypsy* elements, represented 25.7% of the *BAC.SubSample* with a mean length of 1,660 bp. In contrast, 35% of the *Zea* and *Physcomitrella* genomes were made up of *Gypsy* elements with mean lengths of 2,755 and 1,574 bp, respectively. The read-based analyses generally agreed with the repeat proportions of the six analyzed taxa with the exceptions of *Amborella* and *Picea* (Fig. 3c). These two taxa had lower overall repeat proportions in the read-based analyses (58% vs. 25% in *Amborella*, 80% vs. 50% in *Picea*), matching previous similar studies^64^.

We assessed LTR RT richness by comparing recent (>90% LTR similarity) LTR RT exemplars among the seven species compared here (Table 3). *Zea* was by far the most diverse with 4,561 distinct LTR RT exemplars, followed by *Physcomitrella* at 1,217 exemplars and *Picea* with 509. The *CFern v1.1* *A* assembly was low in recent LTR RT diversity with only 22 exemplars, similar to that of *Amborella* and *Marchantia*, which had 11 and 30, respectively. Ancient (75–90% LTR similarity) LTR RT richness differed greatly from recent LTR RT diversity in *Zea* and *Physcomitrella*, which only had 45 and 16 ancient exemplars, respectively (Table 3). *Picea* also had fewer ancient exemplars than recent LTR RTs with 276, but *CFern v1.1* *A* and *Amborella* both had more ancient than recent LTR RT exemplars with 82 and 55, respectively.

**Table 3:** Genome composition and LTR-RT statistics in sampled land plant genomes.

	*Selaginella*	*Marchantia*	*Physcomitrella*	*Amborella*	*Zea*	*Ceratopteris*	*Picea*
Genome Size	0.11	0.2	0.48	0.87	2.1	11.25	20
% GC	45.3	42	33.7	37.5	46.9	37.7	37.6
N50 (Kbp)	1750	1366	17435	4927	217959	22	8
Recent LTR RT	166	30	1217	11	4561	22	509
Ancient LTR RT	33	24	16	55	45	82	276

The quality of the genome assembly could have had a large effect on these interspecific comparisons of repeat diversity, number, and size, as well as genome size, as earlier demonstrated with the *BAC.SubSample*. For example, these eight genome assemblies spanned a 27,000-fold difference in scaffold N50 lengths between that of *Picea* (8 Kbp) and maize (217,960 Kbp). Thus, in addition to the genomes of *CFern v1.1* *A* and *Picea* being many times larger than that of maize, they are much more fragmented, making the identification of repeat elements more difficult and biased for identifying those repeats with smaller lengths.

To investigate LTR RT insertion timing, we identified 62 full-length, high-confidence LTR RT elements in the *CFern v1.1* *A* and *BAC.SubSample* assemblies^65^. The insertion timing of these LTR RTs was relatively uniform over the past 7 million years (Fig. 3d). However, we found considerable differences in the LTR RT identification of these two assemblies as the majority of the identified LTR RTs in the *BAC.SubSample* originated within the last million years, while the *CFern v1.1* *A* assembly did not identify a single LTR RT within the past million years and instead had largely older (>4 mya) LTR RTs. In addition, we note that the *BAC.SubSample* had 28 full-length, high-confidence LTR RTs, while the *CFern v1.1* *A* assembly had 34, despite nearly a 1,000-fold difference in assembly length. These results suggest that the long-read sequencing of the *BAC.SubSample* was able to span and properly assemble these repetitive elements, while the short reads of *CFern v1.1* could only assemble older, more heterogeneous repetitive elements.

## Discussion

Ferns are the second most species-rich clade of vascular plants, with over 10,000 species^50,66^. In addition, ferns are the sister lineage to all seed plants and first appeared approximately 430 million years ago according to fossil-calibrated phylogenies^67^. The oldest unequivocal fossil fern is *Ibyka amphikoma* with a minimum age of 384 million years^67,68^. However, most extant fern diversity arose within the last 40–60 million years during the Cenozoic Era following the rise to dominance of the angiosperms^69,70^. Despite their substantial morphological diversity, sister relationship to seed plants, and lengthy evolutionary history, ferns represent the final frontier of land plant genomics.

Here we provide the first draft genome assembly of the 11.25 Gbp *Ceratopteris* genome, as well as a high-confidence set of gene models. We assessed the role of WGD in the evolutionary history of *Ceratopteris* at three distinct temporal scales. Despite a genome size five times that of classically “large-genome” flowering plants (e.g., maize) and with eight times more chromosomes than *Arabidopsis*, which has undergone at least five WGD events^16^, we found evidence consistent with only one ancient WGD event in *Ceratopteris*. The diploid signal localizations of our BAC-FISH approach refute any recent WGD events that may have been ambiguous in the paralog-age distribution analyses. The three peaks in the paralog-age distribution analyses of *Ceratopteris, Azolla*, and *Equisetum* overlap and thus could potentially be a shared event before the divergence of these three ferns (Fig. 1). However, MAPS analysis indicates that only a minority of subtrees support shared duplications among these three taxa, suggesting three lineage-specific WGD events rather than a single shared event. Based on our analyses and the timing of the WGD event in *Equisetum*, another lineage belonging to the broader fern (monilophyte) clade^57^, the WGD of *Ceratopteris* is likely older than that of *Equisetum* (92 mya) yet younger than the most recent common ancestor of *Ceratopteris* and *Azolla* (~280 mya)^70^ (Fig. 1b). The approaches used here are those standardly used for inferring ancient WGD events from transcriptomic and genomic data. Even complete transcriptomes or chromosome-level genomes can underestimate the true number of WGDs as pseudogenes and gene fragments may be filtered out during analyses. As such, all paralog-age distribution analyses and the resulting inferences regarding WGDs are considered minimum estimates; however, similar analyses of diploid and triploid *Ceratopteris thalictroides* found nearly identical *Ks* frequency distributions to the results presented in this study^71^. If additional WGD events were obscured from the *Ks* analyses due to incomplete gene sampling, it is unlikely that transcriptome samples from a congeneric species would hide the same events. The authors of the above-mentioned study dated the WGD event to 52 ± 1 mya due to their use of a relatively rapid synonymous substitution rate (11.04 × 10^−9^)^71^. Synonymous substitution rates are highly variable among plant lineages and across time^72,73^; thus, we are more confident in our relative dating of the *Ceratopteris* WGD as occurring between that of *Equisetum* and the most recent common ancestor of *Ceratopteris* and *Azolla* due to our MAPS results.

Our results do not support hypotheses of frequent WGD in ferns followed by massive gene silencing and the slow loss of genetic material^74,75^ and instead lend credence to the hypothesis that ferns had ancestrally high chromosome numbers^26,43^ and underwent WGD rarely yet were unable or very slow to lose genetic material^21,31^. This conclusion is in agreement with past studies based on isozymes^25–27^, transcripts of *Equisetum giganteum*^57^, a genetic linkage map of *Ceratopteris*^30^, as well as ancestral reconstructions^76^ that found ancient WGD events to be rare in the evolutionary history of ferns despite the presence of many neopolyploids^32^. While genomic analyses in flowering plants have shown that even very small genomes, such as that of *Arabidopsis*, have undergone numerous rounds of polyploidy, yet still have a low number of chromosomes, we find that ferns are much less dynamic, having undergone relatively few WGD events, yet retaining a high number of chromosomes. Ancestral reconstructions of chromosome numbers across ferns have suggested that the common ancestor of all ferns had a haploid chromosome number of 22, while many of the more diverse fern lineages had higher ancestral chromosome numbers, such as *n* = 30 in Pteridaceae^76^. If chromosome numbers were ancestrally high in ferns, only a single WGD event would therefore be needed to reach a chromosome number of *n* = 39 in *Ceratopteris* (or *n* = 59, the average across all ferns) since the divergence of the common ancestor of ferns from that of seed plants 400 million years ago^67,70^. Alternatively, the high chromosome numbers of ferns could be a result of aneuploidy or chromosomal fission^42^. To better understand the cause of the high chromosome numbers of homosporous ferns, comparative syntenic and phylogenomic analyses will have to be applied across multiple fern taxa based on complete genome assemblies.

Similar to other large plant genomes, a large proportion of the *Ceratopteris* genome is composed of LTR RTs and other transposable elements (Fig. 3). This “genome obesity” is the likely result of a steady accumulation of transposable elements and an inability to discard them, as found in smaller flowering plant genomes^8,40^. Importantly, *Ceratopteris* had a very low diversity of recent LTR RT exemplars when compared to other large-genome species such as maize or *Picea*. While this finding could be indicative of low LTR RT richness and high abundance, given that the counts of the LTR RTs were considerably higher in *Ceratopteris* compared to the other genomes, it is also possible that we are unable to identify the majority of full-length LTR RTs due to low scaffold contiguity with the *CFern v1.1* assembly. The *BAC.SubSample* assembly and read-based analyses provide a more accurate representation of the general repeat composition of *Ceratopteris*; however, these results provide a much smaller representation of the genome and are limited in their resolution. Clearly, long-read technology will be necessary to overcome and fully analyze a genome of this size, as short-read sequencing simply cannot span and assemble the repetitive structures found in *Ceratopteris*. However, expense must be taken into account in any sequencing project, and here the deep sequencing of an 11.25 Gbp genome using long-read technology would take much longer and cost much more than similar sequencing with short reads.

This study provides a major stepping-stone in the understanding of plant evolutionary genomics by providing the first homosporous fern reference genome, as well as unique insights into the processes underlying the formation of these massive genomes. Future efforts should focus on long-read technology to provide a complete assembly of multiple homosporous ferns—thus permitting more extensive comparisons of genome evolution and synteny across green plants.

## Methods

### Tissue samples

*Ceratopteris richardii* (Pteridaceae) is a fast-growing tropical fern, used globally in research laboratories as well as in K-12 and undergraduate biology courses for studying alternation of generations in plants. Inbred lines and single-gene mutants are commercially available and readily produced. For this study, spores from the Hn-n inbred line were kindly donated by Dr. Leslie Hickok (University of Tennessee). The spores were germinated on nutrient media^77,78^ and grown following the recommended conditions in the C-Fern Manual (www.c-fern.org). We isolated the germinated gametophytes to individual petri dishes and growth media. Given that *C. richardii* is homosporous, the gametophytes are typically bisexual and produce both antheridia and archegonia. By isolating the gametophytes prior to sexual maturity, we ensured that any sporophytes that did develop were a product of gametes from a single gametophyte and thus completely homozygous (doubled haploid).

### Library construction and sequencing

We extracted genomic DNA (gDNA) from *Ceratopteris* using a modified CTAB protocol^79^ and quality checked and quantified the gDNA using a Qubit fluorometer (Invitrogen, Carlsbad, CA, USA) and NanoDrop spectrophotometer (Thermo Fisher Scientific, Waltham, MA, USA). Genomic short-read library preparation and sequencing for *Ceratopteris* were completed by the University of Florida’s Interdisciplinary Center for Biotechnology Research (UF ICBR). The gDNA was fragmented and size-selected for ~300 base pair (bp) inserts, and the sequencing of 150 bp paired-end (PE) reads was conducted on two runs of the Illumina NextSeq platform (Illumina, San Diego, CA, USA). Mate-pair (MP) libraries (125 bp PE, 8–10 Kbp inserts) were prepared and sequenced at the Duke Genome Sequencing and Analysis Core on two lanes of Illumina HiSeq 2000 (Illumina, San Diego, CA, USA).

We also subsampled the *Ceratopteris* genome with long-read technology to avoid the assembly biases inherent in short-read technology by sequencing BAC clones (Plate CR_Ba #624, Green Plant BAC Library Project, provided by Clemson University Genomics Institute). We selected 34 *Ceratopteris* Hn-n BAC clones to be grown, pooled, purified, and sequenced using the RS II platform (Pacific Bioscience, Menlo Park, CA, USA) at the Arizona Genomics Institute. The reads were cleaned and *de novo* assembled using the Hierarchical Genome Assembly Process (HGAP) in the SMRT Analysis software package (Pacific Biosciences, Menlo Park, CA, USA) to produce the *BAC.SubSample* assembly.

Long-read technology was also used to acquire a high-confidence set of gene models from sporophyte tissue. We extracted total RNA from sexually mature leaf tissue using the RNeasy Plant Mini kit (Qiagen, Hilden, Germany). The total RNA was size-selected for 0.8–2, 2–3, 3–5, and >5 Kbp with the SageELF (Sage Science, Beverly, MA, USA) at the UF ICBR. The libraries were prepared following the SMRTbell library protocol, and each library was sequenced on three PacBio SMRT cells (Pacific Bioscience, Menlo Park, CA, USA) at the UF ICBR.

### Genome assembly

The raw genomic PE reads were trimmed of adapters and then quality-filtered with Trimmomatic^80^, while the raw MP reads were trimmed of adapters and separated into MP, PE, and unknown reads with NxTrim^81^. All libraries were quality-checked before and after cleaning with FastQC (www.bioinformatics.babraham.ac.uk/projects/fastqc/). We divided the cleaned PE reads into 24-mers with Jellyfish^82^ and plotted their frequencies with KAT^83^ to assess environmental contamination, organellar genome content, nuclear genome size, and repeat content.

The PE reads were assembled using Meraculous2^84^ and a k-mer size of 61 based on the results of KmerGenie^85^ to produce assembly *CFern v1.0*. The scaffolds from the *CFern v1.0* assembly were further scaffolded with the MP reads using the SSPACE assembler^86^ to produce the genome assembly *CFern v1.1*. To compare the content of *CFern v1.1* with the overall content of the cleaned reads, we divided the assembly into 24-mers with Jellyfish^82^ and compared the resulting frequencies to those of the cleaned PE reads using the compare feature of KAT^83^. For subsequent analyses, only scaffolds over 10 Kbp were used (*CFern v1.1A*).

### Transcriptome assembly

We cleaned and processed the long reads following the IsoSeq protocol^54^ in which the circular consensus sequences (CCS) were acquired from the raw reads and then classified and clustered. Only full-length, high-quality (accuracy >= 99%), polished sequences (*IsoSeq.HQ*) were used for analysis following the Iterative Clustering and Error correction (ICE)/Quiver algorithm. The *IsoSeq.HQ* sequences were further collapsed into unique isoforms and genes using both genome-based and sequence-based protocols (see below).

For the genome-based method, the *IsoSeq.HQ* sequences were mapped to the *CFern v1.1A* assembly using GMAP (parameters: -f samse –n 0 –z sense_force)^87^. The sam file output was sorted (parameters: -k 3,3 –k 4,4n), and transcripts were collapsed together (collapse_isoforms_by_sam.py, https://github.com/Magdoll/cDNA_Cupcake)^54^. We used both 98% coverage and 98% identity as our full-length mapping cutoff and then searched for incomplete genes with 50% coverage and 98% identity.

Due to the fragmented state of the *CFern v1.1A* assembly, many transcripts did not map. Thus, we also used CD-Hit v4.6.4 (parameters: -c 0.99 –G 0 –aL 0.90 –AL 100 –aS 0.99 –AS 30)^88^ to cluster and collapse highly similar transcripts into putative isoforms without a reference genome. We then used those sequences with the Coding Genome reconstruction tool^89^ for genome-free isoform collapse and gene identification. This pipeline divided the sequences into 30-mers and then grouped those kmers into clusters based on pairwise distances. De Bruijn graphs of the sequences for each cluster were then used to resolve sequencing errors and alternative splicing events and output putative genes. Due to the high accuracy, full-length, *de novo* nature of IsoSeq and subsequent cleaning protocols, these genes served as our reference gene models for *Ceratopteris* (referred to as *UniCFernModels*).

### Polyploidy

The *UniCFernModels* data set was used in the DupPipe pipeline^90,91^ to estimate the relative age of gene duplications. DupPipe finds duplicate gene pairs and then estimates the divergence of these genes using the number of substitutions per synonymous site (*K*_*S*_). The frequency of duplicate genes corresponding to a given level of divergence, as a substitute for timing, was plotted as a histogram, and peaks were inferred to represent synchronous gene duplications, indicative of ancient polyploidy events^90,92^. Genes from two other ferns, *Equisetum giganteum*^57^ and *Azolla filiculoides*^19^, were similarly analyzed and plotted for comparison. To reduce the subjectivity of smoothing based on varying bin sizes, we analyzed the *K*_*S*_ values of these three ferns using the SiZer (Significance of Zero Crossings of the Derivative)^93^ package in R v3.4.2^94^. This analysis determines whether an increase or decrease in a scatterplot or histogram is significant at α = 0.05 and plots the changes along the original *x*-axis with blue coloration indicating a significant increase, red a significant decrease, purple insignificance, and gray too few data points to determine.

To determine whether the three ferns examined here (*Ceratopteris*, *Equisetum*, and *Azolla*), spanning over 400 million years since their most recent common ancestor^70^, share any ancient polyploidy events, we clustered the predicted proteins of *Ceratopteris, Equisetum, Azolla, Amborella, Selaginella*, and *Physcomitrella* into orthogroups using OrthoFinder^95^. Only orthogroups with gene representatives from all six species were retained. The protein sequences of each orthogroup were aligned with MAFFT^96^, and the alignments were converted to nucleotide alignments using the pxaa2cdn tool in Phyx^97^. The alignments were stripped of highly ambiguous (>90% missing data) columns, and gene trees were produced with RAxML using 100 rapid bootstrap searches and the GTRGAMMA model of evolution^98^. These gene family trees were entered into the Multi-tAxon Paleopolyploidy Search (MAPS) package^58^. This package first filters all of the gene family trees for subtrees that match the known species tree [here (*Physcomitrella*, (*Selaginella*, (*Amborella*, (*Equisetum*, (*Ceratopteris*, *Azolla*)))))]. It then counts the number of subtrees with gene duplications at a specific node in the species tree relative to the number of available subtrees. A node with a high proportion of gene duplications is presumed to have a shared polyploidy event.

We also used a cytogenetic approach to assess more recent WGD. We conducted fluorescent *in situ* hybridization (FISH) using the previously described BAC clones as probes following Chester *et al*.^99^ and Chamala *et al*.^100^. To produce the probes, the BAC DNA was extracted from the *Escherichia coli* culture and amplified by rolling circle amplification (RCA)^101^. The RCA product was labeled by nick translation with Cy5-dUTP and purified with a QIAquick Nucleotide Removal kit (Qiagen, Venlo, Netherlands).

Root tips for chromosome preparations were collected in the mornings and immediately treated with pressurized nitrous oxide for 1 hour before being fixed in 3:1 ethanol (EtOH): glacial acetic acid overnight at room temperature and transferred to 70% EtOH at −20 °C for long-term storage. The root tips were then treated and chromosome spreads prepared to produce slides for *in situ* hybridization with the fluorescently labeled probes^99^. The BAC FISH images were taken on an AxioImager M2 microscope with an AxioCam MR camera (Carl Zeiss AG, Oberkochen, Germany).

### Repeat characterization

We took both structural- and homology-based approaches to repeat characterization following Campbell *et al*.^102^. As long terminal repeat retrotransposons (LTR RTs) comprise a sizable proportion of most plant genomes, a variety of tools was used to characterize these repeats in the *CFern v1.1A* assembly. Recent LTR RTs were collected based on 90% LTR similarity using LTRharvest (parameters: -minlenltr 100 -maxlenltr 6000 -mindistltr 1500 -maxdistltr 25000 -mintsd 5 -maxtsd 5 -motif tgca -similar 90 -vic 10)^103^ from the GenomeTools package^104^. LTRdigest was then used to find elements with poly purine tracts (PPT) or primer binding sites (PBS) using the Genomic tRNA database^105^. Those elements were identified and further filtered for false positive elements by removing gappy elements (>50 Ns), recent gene duplications where the flanking regions of the LTRs are alignable, and nested RT insertions using custom scripts. LTR RTs with nested DNA transposons were also identified by searching DNA transposase protein sequences with BLASTx^106^. LTR RT exemplars were then identified based on 80% identity and 90% coverage from the filtered elements based on the internal sequences of the LTR RTs and then based on the LTR sequences. Older LTR RTs were similarly collected but with 75% similarity among the LTR sequences and lacking the TGCA motif. To exclude more recent LTR RTs from the older LTR RT library, the younger LTR RT exemplars were used to mask and exclude elements found in the older LTR RT library with RepeatMasker^107^. The two LTR RT libraries were combined (*allLTR.lib*) and used as the reference library to mask the *CFern v1.1A* assembly with RepeatMasker^107^.

The unmasked remainder of the assembly was inputted into RepeatModeler to identify repeat families *de novo*^108^. The RepeatModeler library and LTR RT library were combined, and unidentified repeats were searched against a transposase database^107,109^ using BLASTx and identified to superfamily when possible^106^. To ensure that fragmented plant genes were not included in the final repeat library, we queried all of our repeats with the SwissProt plant protein^110^ and NCBI RefSeq plant protein databases using BLASTx^106^. With our clean, final repeat library, we used RepeatMasker to quantify the repeat elements throughout *CFern v1.1A*.

To make direct comparisons with other plant genome assemblies of varying sizes, qualities, and lineages, we followed the same repeat annotation protocol for the genomes of *Amborella trichopoda*^16^, a monocot (*Zea mays*)^60^, a liverwort (*Marchantia polymorpha*)^61^, a lycophyte (*Selaginella moellendorffii*)^62^, a conifer (*Picea abies*)^8^, and a moss (*Physcomitrella patens*)^63^. We also ran the same protocol on the *BAC.SubSample* assembly. To remove assembly biases, we used RepeatExplorer 2^111^ on cleaned reads for the above-mentioned taxa, with the exception of *Selaginella* which only used Sanger sequencing. Raw reads were downloaded from the NCBI Sequence Read Archive and EMBL European Nucleotide Archive, cleaned and reduced to 0.5X coverage of their respective genomes, then run in RepeatExplorer2^111^ via the Elixir CZ Galaxy portal under default parameters against the Viridiplantae version 3.0 transposable element protein domain database.

### Dating repeat insertion events

We used the highly accurate but conservative LTR_Retriever package^65^ to identify full-length LTR RTs and date their insertion using both the *CFern v1.1A* and *BAC.SubSample* assemblies. We provided candidate LTR RTs from LTR_harvest and LTR_finder using a 90% similarity minimum threshold between LTRs and the presence of the TGCA motifs. The candidate LTR RTs were filtered, removing non-LTR RT repeat elements or those with large amounts of tandem repeats or gaps. Especially in fragmented genome assemblies, such as the *CFern v1.1A*, these requirements hugely reduce the number of LTR RT candidates but ensure that only full-length LTR RTs are analyzed. Following filtering, the long terminal repeat regions of each transposable element were aligned, and the Jukes-Cantor model was used to estimate the divergence time of the two LTR regions. We used a mutation rate of 6.5 × 10^−9^ per site per year to estimate the years since insertion^16^. This mutation rate is half that of rice^36^ and is a general estimate; therefore, the insertion times should only be used in reference to the relative timing of insertion, rather than as exact dates.

## Data Availability

All of the raw reads and associated genomic and transcriptomic assemblies can be found in the NCBI BioProjects under PRJNA511033. All of the tissue used for sequencing came from one doubled haploid genotype (Voucher: M. Whitten #5841, University of Florida Herbarium, FLAS).

## References

[1] Lughadha EN (2016). Counting counts: Revised estimates of numbers of accepted species of flowering plants, seed plants, vascular plants and land plants with a review of other recent estimates. Phytotaxa.

[2] Bennett, M. D. & Leitch, I. J. Plant DNA C-values database (release 6.0, Dec. 2012). *WWW Doc. URL http//data.kew.org/cvalues/*. *[accessed 14 Oct. 2014]* (2012).

[3] Rice A (2015). The Chromosome Counts Database (CCDB) – a community resource of plant chromosome numbers. New Phytol..

[4] Greilhuber J (2006). Smallest angiosperm genomes found in Lentibulariaceae, with chromosomes of bacterial size. Plant Biol..

[5] Pellicer J, Fay MF, Leitch IJ (2010). The largest eukaryotic genome of them all?. Bot. J. Linn. Soc..

[6] Ghatak J (1977). Biosystematic survey of pteridophytes from Shevaroy Hills, south India. Nucleus.

[7] Bennett MD (1998). Plant genome values: How much do we know?. Proc. Natl. Acad. Sci..

[8] Nystedt B (2013). The Norway spruce genome sequence and conifer genome evolution. Nature.

[9] Zimin A (2014). Sequencing and assembly of the 22-gb loblolly pine genome. Genetics.

[10] Birol, I. *et al*. Assembling the 20 Gb white spruce (*Picea glauca*) genome from whole-genome shotgun sequencing data. *Bioinforma*. 10.1093/bioinformatics/btt178 (2013). 10.1093/bioinformatics/btt178 PMC3673215 23698863

[11] Stebbins GL (1940). The significance of polyploidy in plant evolution. Am. Nat..

[12] Grant, V. Plant Speciation (Columbia University Press, 1981).

[13] Jiao Y (2012). A genome triplication associated with early diversification of the core eudicots. Genome Biol..

[14] Tang H, Bowers JE, Wang X, Paterson AH (2010). Angiosperm genome comparisons reveal early polyploidy in the monocot lineage. Proc. Natl. Acad. Sci..

[15] Jiao Y (2011). Ancestral polyploidy in seed plants and angiosperms. Nature.

[16] *Amborella* Genome Project. The *Amborella* Genome and the Evolution of Flowering Plants. *Science (80-.)*, **342**, (2013). 10.1126/science.1241089 24357323

[17] Landis, J. B. *et al*. Impact of whole‐genome duplication events on diversification rates in angiosperms. *Am. J. Bot*. (2018). 10.1002/ajb2.1060 29719043

[18] Carretero-Paulet L (2015). High Gene Family Turnover Rates and Gene Space Adaptation in the Compact Genome of the Carnivorous Plant Utricularia gibba. Mol. Biol. Evol..

[19] Li, F.-W. *et al*. Fern genomes elucidate land plant evolution and cyanobacterial symbioses. *Nat. plants*, 1 (2018). 10.1038/s41477-018-0188-8 PMC6786969 29967517

[20] Klekowski E, Baker H (1966). Evolutionary Significance of Polyploidy in the Pteridophyta. Science (80-.)..

[21] Haufler CH (1987). Electrophoresis is Modifying Our Concepts of Evolution in Homosporous Pteridophytes. Am. J. Bot..

[22] Wagner, W. H. & Wagner, F. S. Polyploidy in pteridophytes. In *Polyploidy* 199–214 (Springer, 1980).

[23] Klekowski E (1972). Genetical features of ferns as contrasted with seed plants. Ann. Missouri Bot. Gard..

[24] Haufler CH (2016). Sex and the single gametophyte: Revising the homosporous vascular plant life cycle in light of contemporary research. Bioscience.

[25] Soltis, D. E. Genetic evidence for diploidy in *Equisetum*. *Am. J. Bot*., 908–913 (1986).

[26] Haufler, C. H. & Soltis, D. E. Evolutionary Significance of Polyploidy in the Pteridophyta., **83**, 4389–4393 (1986).

[27] Soltis, P. S. & Soltis, D. E. Electrophoretic evidence for genetic diploidy in *Psilotum nudum*. *Am. J. Bot*., 1667–1671 (1988).

[28] Pichersky E, Soltis D, Soltis P (1990). Defective chlorophyll a/b-binding protein genes in the genome of a homosporous fern. Proc. Natl. Acad. Sci..

[29] Soltis, D. E. & Soltis, P. S. The distribution of selfing rates in homosporous ferns. *Am. J. Bot*. (1992).

[30] Nakazato T, Jung M-K, Housworth EA, Rieseberg LH, Gastony GJ (2006). Genetic map-based analysis of genome structure in the homosporous fern *Ceratopteris richardii*. Genetics.

[31] Barker, M. S. Evolutionary Genomic Analyses of Ferns Reveal that High Chromosome Numbers are a Product of High Retention and Fewer Rounds of Polyploidy Relative to Angiosperms. *Am. Fern J*., **99**, 136–141 CR-Copyright © 2009 American Fern (2009).

[32] Wood TE (2009). The frequency of polyploid speciation in vascular plants. Proc. Natl. Acad. Sci..

[33] Nakazato, T., Barker, M. S., Rieseberg, L. H. & Gastony, G. J. Evolution of the nuclear genome of ferns and lycophytes. In *Biology and evolution of ferns and lycophytes* (Cambridge University Press, 2008).

[34] Sessa, E. B. & Der, J. P. Evolutionary genomics of ferns and lycophytes. In *Advances in Botanical Research* **78**, 215–254 (Elsevier, 2016).

[35] Piegu B (2006). Doubling genome size without polyploidization: Dynamics of retrotransposition-driven genomic expansions in *Oryza australiensis*, a wild relative of rice. Genome Res..

[36] Ma J, Bennetzen JL (2004). Rapid recent growth and divergence of rice nuclear genomes. Proc. Natl. Acad. Sci. USA.

[37] Vitte C, Bennetzen JL (2006). Analysis of retrotransposon structural diversity uncovers properties and propensities in angiosperm genome evolution. Proc. Natl. Acad. Sci..

[38] Estep MC, DeBarry JD, Bennetzen JL (2013). The dynamics of LTR retrotransposon accumulation across 25 million years of panicoid grass evolution. Heredity (Edinb)..

[39] Bennetzen JL, Wang H (2014). The contributions of transposable elements to the structure, function, and evolution of plant genomes. Annu. Rev. Plant Biol..

[40] Devos KM, Brown JKM, Bennetzen JL (2002). Genome Size Reduction through Illegitimate Recombination Counteracts Genome Expansion in *Arabidopsis*. Genome Res..

[41] Hawkins JS, Kim H, Nason JD, Wing RA, Wendel JF (2006). Differential lineage-specific amplification of transposable elements is responsible for genome size variation in Gossypium. Genome Res..

[42] De La Torre AR (2014). Insights into conifer giga-genomes. Plant Physiol..

[43] Soltis DE, Soltis PS (1987). Polyploidy and Breeding Systems in Homosporous Pteridophyta: A Reevaluation. Am. Nat..

[44] Sessa, E. B. *et al*. Between Two Fern Genomes. *Gigascience*, **3**, (2014). 10.1186/2047-217X-3-15 PMC4199785 25324969

[45] Durand LZ, Goldstein G (2001). Photosynthesis, photoinhibition, and nitrogen use efficiency in native and invasive tree ferns in Hawaii. Oecologia.

[46] Ellwood MDF, Foster Wa (2004). Doubling the estimate of invertebrate biomass in a rainforest canopy. Nature.

[47] Fayle TM, Chung AYC, Dumbrell AJ, Eggleton P, Foster WA (2009). The Effect of Rain Forest Canopy Architecture on the Distribution of Epiphytic Ferns (*Asplenium spp*.) in Sabah, Malaysia. Biotropica.

[48] Paul B (2014). Azolla domestication towards a biobased economy?. New Phytol..

[49] Shukla AK (2016). Expression of an insecticidal fern protein in cotton protects against whitefly. Nat. Biotechnol..

[50] PPG I (2016). A community derived classification for extant lycophytes and ferns. J. Syst. Evol..

[51] Pryer KM, Schneider H, Zimmer EA, Ann Banks J (2002). Deciding among green plants for whole genome studies. Trends Plant Sci..

[52] Soltis PS, Soltis DE (2013). A conifer genome spruces up plant phylogenomics. Genome Biol..

[53] Warren WC (2008). Genome analysis of the platypus reveals unique signatures of evolution. Nature.

[54] Gordon SP (2015). Widespread polycistronic transcripts in fungi revealed by single-molecule mRNA sequencing. PLoS One.

[55] Simão FA, Waterhouse RM, Ioannidis P, Kriventseva EV, Zdobnov EM (2015). BUSCO: assessing genome assembly and annotation completeness with single-copy orthologs. Bioinformatics.

[56] Cui L (2006). Widespread genome duplications throughout the history of flowering plants. Genome Res..

[57] Vanneste, K., Sterck, L., Myburg, Z., Van de Peer, Y. & Mizrachi, E. Horsetails Are Ancient Polyploids: Evidence from *Equisetum giganteum*. *Plant Cell*, 1–13 10.1105/tpc.15.00157 (2015). 10.1105/tpc.15.00157 PMC4498207 26002871

[58] Li, Z. *et al*. Early genome duplications in conifers and other seed plants. *Sci. Adv*., **1**, (2015). 10.1126/sciadv.1501084 PMC4681332 26702445

[59] Lim KARY, Matyaksek R, Kovarik A, Leitch AR (2004). Genome evolution in allotetraploid. Nicotiana. Biol. J. Linn. Soc..

[60] Hirsch, C. *et al*. Draft assembly of elite inbred line PH207 provides insights into genomic and transcriptome diversity in maize. *Plant Cell* tpc-00353 (2016). 10.1105/tpc.16.00353 PMC5155341 27803309

[61] Bowman JL (2017). Insights into land plant evolution garnered from the *Marchantia polymorpha* genome. Cell.

[62] Banks JA (2011). The *Selaginella* Genome Identifies Genetic Changes Associated with the Evolution of Vascular. Plants. Sci..

[63] Lang D (2018). The *Physcomitrella patens* chromosome‐scale assembly reveals moss genome structure and evolution. Plant J..

[64] Wolf, P. G. *et al*. An exploration into fern genome space. *Genome Biol. Evol*. 10.1093/gbe/evv163 (2015). 10.1093/gbe/evv163 PMC4607520 26311176

[65] Ou S, Jiang N (2018). LTR_retriever: A Highly Accurate and Sensitive Program for Identification of Long Terminal Repeat Retrotransposons. Plant Physiol..

[66] Smith AR (2006). A classification for extant ferns. Taxon.

[67] Morris JL (2018). The timescale of early land plant evolution. Proc. Natl. Acad. Sci..

[68] Skog JE, Banks HP (1973). *Ibyka amphikoma*, gen. et sp. n., a new protoarticulate precursor from the late Middle Devonian of New York State. Am. J. Bot..

[69] Schuettpelz E, Pryer KM (2007). Fern phylogeny inferred from 400 leptosporangiate species and three plastid genes. Taxon.

[70] Testo W, Sundue M (2016). A 4000-species dataset provides new insight into the evolution of ferns. Mol. Phylogenet. Evol..

[71] Zhang R (2019). Dating Whole Genome Duplication in *Ceratopteris thalictroides* and Potential Adaptive Values of Retained Gene Duplicates. Int. J. Mol. Sci..

[72] De La Torre AR, Li Z, Van de Peer Y, Ingvarsson PK (2017). Contrasting rates of molecular evolution and patterns of selection among gymnosperms and flowering plants. Mol. Biol. Evol..

[73] Bromham L, Hua X, Lanfear R, Cowman PF (2015). Exploring the relationships between mutation rates, life history, genome size, environment, and species richness in flowering plants. Am. Nat..

[74] Haufler CH (2002). Homospory 2002: An Odyssey of progress in pteridophyte genetics and evolutionary biology: Ferns and other homosporous vascular plants have highly polyploid chromosome numbers, but they express traits following diploid models and, although capable of extre. AIBS Bull..

[75] Haufler CH (2014). Ever since Klekowski: testing a set of radical hypotheses revives the genetics of ferns and lycophytes. Am. J. Bot..

[76] Clark J (2016). Genome evolution of ferns: evidence for relative stasis of genome size across the fern phylogeny. New Phytol..

[77] Bold, H. C. Morphology of Plants (Harper and Row, 1957).

[78] Nitsch, J. P. Growth and development *in vitro* of excised ovaries. *Am. J. Bot*., 566–577 (1951).

[79] Doyle J, Doyle JL (1987). Genomic plant DNA preparation from fresh tissue-CTAB method. Phytochem Bull.

[80] Bolger AM, Lohse M, Usadel B (2014). Trimmomatic: a flexible trimmer for Illumina sequence data. Bioinformatics.

[81] O’Connell J (2015). NxTrim: optimized trimming of Illumina mate pair reads. Bioinformatics.

[82] Marçais G, Kingsford C (2011). A fast, lock-free approach for efficient parallel counting of occurrences of k-mers. Bioinformatics.

[83] Mapleson D, Garcia Accinelli G, Kettleborough G, Wright J, Clavijo BJ (2016). KAT: a K-mer analysis toolkit to quality control NGS datasets and genome assemblies. Bioinformatics.

[84] Chapman, J. A., Ho, I. Y., Goltsman, E. & Rokhsar, D. S. Meraculous2: fast accurate short-read assembly of large polymorphic genomes. *arXiv Prepr. arXiv1608.01031* (2016).

[85] Chikhi R, Medvedev P (2014). Informed and automated k-mer size selection for genome assembly. Bioinformatics.

[86] Boetzer M, Henkel CV, Jansen HJ, Butler D, Pirovano W (2010). Scaffolding pre-assembled contigs using SSPACE. Bioinformatics.

[87] Wu TD, Watanabe CK (2005). GMAP: a genomic mapping and alignment program for mRNA and EST sequences. Bioinformatics.

[88] Fu L, Niu B, Zhu Z, Wu S, Li W (2012). CD-HIT: accelerated for clustering the next-generation sequencing data. Bioinformatics.

[89] Workman RE (2018). Single-molecule, full-length transcript sequencing provides insight into the extreme metabolism of the ruby-throated hummingbird *Archilochus colubris*. Gigascience.

[90] Barker MS (2008). Multiple paleopolyploidizations during the evolution of the Compositae reveal parallel patterns of duplicate gene retention after millions of years. Mol. Biol. Evol..

[91] Barker MS (2010). EvoPipes. net: bioinformatic tools for ecological and evolutionary genomics. Evol. Bioinforma..

[92] Lynch M, Conery JS (2000). The Evolutionary Fate and Consequences of Duplicate. Genes. Sci..

[93] Chaudhuri P, Marron JS (1999). SiZer for exploration of structures in curves. J. Am. Stat. Assoc..

[94] R Core Team. R: A language and environment for statistical computing. (2013).

[95] Emms DM, Kelly S (2015). OrthoFinder: solving fundamental biases in whole genome comparisons dramatically improves orthogroup inference accuracy. Genome Biol..

[96] Katoh K, Standley DM (2013). MAFFT multiple sequence alignment software version 7: improvements in performance and usability. Mol. Biol. Evol..

[97] Brown JW, Walker JF, Smith SA (2017). Phyx: phylogenetic tools for unix. Bioinformatics.

[98] Stamatakis A (2014). RAxML version 8: a tool for phylogenetic analysis and post-analysis of large phylogenies. Bioinforma..

[99] Chester M (2012). Extensive chromosomal variation in a recently formed natural allopolyploid species, *Tragopogon miscellus* (Asteraceae). Proc. Natl. Acad. Sci..

[100] Chamala S (2013). Assembly and validation of the genome of the nonmodel basal angiosperm *Amborella*. Science.

[101] Berr A, Schubert I (2006). Direct labelling of BAC DNA by rolling circle amplification. Plant J..

[102] Campbell MS (2014). MAKER-P: a tool kit for the rapid creation, management, and quality control of plant genome annotations. Plant Physiol..

[103] Ellinghaus D, Kurtz S, Willhoeft U (2008). LTRharvest, an efficient and flexible software for de novo detection of LTR retrotransposons. BMC Bioinformatics.

[104] Gremme G, Steinbiss S, Kurtz S (2013). GenomeTools: a comprehensive software library for efficient processing of structured genome annotations. IEEE/ACM Trans. Comput. Biol. Bioinforma..

[105] Chan PP, Lowe TM (2015). GtRNAdb 2.0: an expanded database of transfer RNA genes identified in complete and draft genomes. Nucleic Acids Res..

[106] Camacho C (2009). BLAST+: architecture and applications. BMC Bioinformatics.

[107] Smith, A. F. A., Hubley, R. & Green, P. RepeatMasker Open-4.0. 2013–2015.

[108] Smit, A. F. A. & Hubley, R. RepeatModeler Open-1.0. *Available fom http//www.repeatmasker.org* (2008).

[109] Kennedy RC, Unger MF, Christley S, Collins FH, Madey GR (2011). An automated homology-based approach for identifying transposable elements. BMC Bioinformatics.

[110] Schneider M (2009). The UniProtKB/Swiss-Prot knowledgebase and its Plant Proteome Annotation Program. J. Proteomics.

[111] Novák P, Neumann P, Pech J, Steinhaisl J, Macas J (2013). RepeatExplorer: a Galaxy-based web server for genome-wide characterization of eukaryotic repetitive elements from next-generation sequence reads. Bioinformatics.

